# Novel Mechanism by a Bis-Pyridinium Fullerene Derivative to Induce Apoptosis by Enhancing the MEK-ERK Pathway in a Reactive Oxygen Species-Independent Manner in BCR-ABL-Positive Chronic Myeloid Leukemia-Derived K562 Cells

**DOI:** 10.3390/ijms23020749

**Published:** 2022-01-11

**Authors:** Kazuya Sumi, Kenji Tago, Yosuke Nakazawa, Kyoko Takahashi, Tomoyuki Ohe, Tadahiko Mashino, Megumi Funakoshi-Tago

**Affiliations:** 1 Division of Hygienic Chemistry, Faculty of Pharmacy, Keio University, 1-5-30 Shibakoen, Minato-ku, Tokyo 105-8512, Japan; kazuya.sumi@keio.jp (K.S.); nakazawa-ys@pha.keio.ac.jp (Y.N.); 2 Division of Structural Biochemistry, Department of Biochemistry, Jichi Medical University, Shimotsuke 321-0498, Japan; 3 Division of Pharmaceutical Sciences, Faculty of Pharmacy, Keio University, 1-5-30 Shibakoen, Minato-ku, Tokyo 105-8512, Japan; kyoko-takahashi@nms.ac.jp (K.T.); ohe-tm@pha.keio.ac.jp (T.O.); mashino-td@keio.jp (T.M.)

**Keywords:** bis-pyridinium fullerene derivative (BPF), BCR-ABL, chronic myeloid leukemia (CML), MEK-ERK pathway, reactive oxygen species (ROS)

## Abstract

In the treatment of breakpoint cluster region-Abelson (BCR-ABL)-positive chronic myeloid leukemia (CML) using BCR-ABL inhibitors, the appearance of a gatekeeper mutation (T315I) in BCR-ABL is a serious issue. Therefore, the development of novel drugs that overcome acquired resistance to BCR-ABL inhibitors by CML cells is required. We previously demonstrated that a bis-pyridinium fullerene derivative (BPF) induced apoptosis in human chronic myeloid leukemia (CML)-derived K562 cells partially through the generation of reactive oxygen species (ROS). We herein show that BPF enhanced the activation of the mitogen-activated protein kinase/extracellular signal-regulated kinase kinase-extracellular signal-regulated kinase (MEK-ERK) pathway in a ROS-independent manner. BPF-induced apoptosis was attenuated by trametinib, suggesting the functional involvement of the MEK-ERK pathway in apoptosis in K562 cells. In addition, the constitutive activation of the MEK-ERK pathway by the enforced expression of the BRAFV600E mutant significantly increased the sensitivity of K562 cells to BPF. These results confirmed for the first time that BPF induces apoptosis in K562 cells through dual pathways—ROS production and the activation of the MEK-ERK pathway. Furthermore, BPF induced cell death in transformed Ba/F3 cells expressing not only BCR-ABL but also T315I mutant through the activation of the MEK-ERK pathway. These results indicate that BPF is as an effective CML drug that overcomes resistance to BCR-ABL inhibitors.

## 1. Introduction

Chronic myeloid leukemia (CML) is a clonal myeloproliferative disease that accounts for 15–20% of adult leukemias [1]. The majority of patients with CML have a reciprocal translocation of the t (9; 22) (q34; q11) chromosome, which causes the appearance of the Philadelphia chromosome (Ph) [2]. The appearance of Ph results in the fusion of the sequence encoding the breakpoint cluster region (BCR) with the region encoding the ABL tyrosine kinase, and the fused gene *Bcr-Abl* is expressed [3]. The fusion protein BCR-ABL is a tyrosine kinase that is constitutively activated by oligomerization and subsequent autophosphorylation [4]. BCR-ABL phosphorylates the transcription factor signal transducer and activator of transcription 5 (STAT5) at tyrosine 694 (Y694) and activates it. STAT5 functions as a major tumorigenesis driver by inducing the expression of anti-apoptotic proteins and factors inducing cell proliferation [5,6]. In addition, the phosphorylation of the tyrosine residue in BCR-ABL at 117 (Y177) recruits the adaptor protein GRB2, and this is followed by the binding of SOS. The protein complex including BCR-ABL/GRB2/SOS induces the conversion of Ras from the inactive GDP-bound form to the active GTP-bound form, and this, in turn, activates the Raf/MEK/ERK pathway, which is involved in cell survival and proliferation [7,8,9].

The development of BCR-ABL inhibitors, such as imatinib, nilotinib, and dasatinib, has markedly improved the treatment of CML [10,11,12]. However, the continued administration of these BCR-ABL inhibitors leads to the development of secondary mutations in the *Bcr-Abl* gene. One of the most frequent mutations is the substitution of threonine at 315 to isoleucine (T315I) in BCR-ABL, called the gatekeeper mutation. The introduction of the T315I mutation prevents the binding of BCR-ABL to three types of BCR-ABL inhibitors [13,14,15]. Therefore, the development of novel drugs that overcome gatekeeper mutation-induced acquired resistance to BCR-ABL inhibitors by CML cells is needed for the treatment of patients with CML.

We have been conducting research aimed at applying fullerene (C_60_), a carbon nanostructure, to anti-cancer drugs. We previously reported that cationic fullerene derivatives exerted anti-proliferative effects through the generation of reactive oxygen species (ROS) in a wide range of human cancer cell lines, including solid cancers and hematological malignancies [16,17,18,19]. We recently demonstrated that a bis-pyridinium fullerene derivative (BPF), one of the fullerene derivatives into which a pyridinium group was introduced, exhibit potent cytotoxicity to CML-derived K562 cells. We also observed that BPF induced activation of caspase-3, caspase-8, and caspase-9 in K562 cells, indicating that BPF induces both extrinsic and intrinsic apoptotic pathways [20]. BPF exhibited markedly lower cytotoxicity against untransformed peripheral blood mononuclear cells (PBMCs) than K562 cells. Interestingly, we found that BPF induced the downregulation of BCR-ABL through transcriptional suppression and protein degradation. Both of these suppressive machineries are triggered by ROS production. However, when ROS generation induced by BPF was prevented by the antioxidant edaravone, the downregulation of BCR-ABL was significantly canceled but apoptosis in K562 cells were partially inhibited [20]. These results suggest the possibility that some BPF-treated K562 cells died due to ROS-independent apoptosis.

Therefore, we hypothesized that BPF induced apoptosis in K562 cells in both ROS-dependent and ROS-independent unknown manners. In the present study, we found that BPF enhanced activation of the MEK-ERK pathway in K562 cells in a ROS-independent manner. Therefore, we aimed to investigate whether the enhancement of MEK-ERK pathway contributes to BPF-induced apoptosis of K562 cells by using trametinib, which is a MEK inhibitor. We also aimed to investigate whether BPF induces apoptosis via MEK-ERK pathway in Ba/F3 cells transformed with the BCR-ABL T315I mutant that exhibits resistance to BCR-ABL inhibitors and to verify that BPF is an effective CML therapeutic agent with a novel mechanism.

## 2. Results

### 2.1. BPF Enhanced the Activation of the MEK-ERK Pathway in K562 Cells

**Figure 1:**
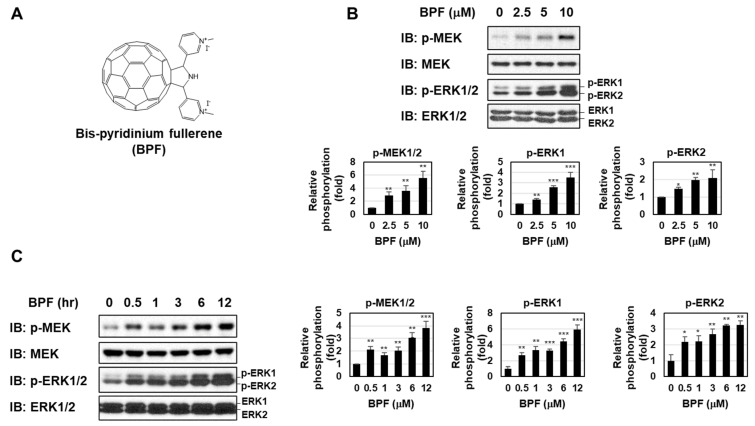
A bis-pyridinium fullerene derivative enhanced the activation of the MEK-ERK pathway in K562 cells. (**A**) Structure of the bis-pyridinium fullerene derivative (BPF). (**B**) K562 cells were treated with BPF (2.5, 5, and 10 μM) for 6 h. (**C**) K562 cells were treated with BPF (5 μM) for the indicated periods. (**B**,**C**) The phosphorylation and/or expression of MEK and ERK were detected by immunoblotting. The relative phosphorylation levels of MEK, ERK1, and ERK2 are shown in the graphs. * *p* < 0.05, ** *p* < 0.01, and *** *p* < 0.001 indicate a significant difference from control cells.

We previously showed that BPF, one of the fullerene derivatives into which a pyridinium group was introduced (Figure 1A), potently induced apoptosis in CML-derived K562 cells partially through the generation of ROS [20]. When K562 cells were treated with BPF for 6 h, the phosphorylation of MEK and ERK was markedly enhanced in a dose-dependent manner (Figure 1B). Furthermore, the enhanced activation of the MEK-ERK pathway by BPF was sustained for at least 12 h in K562 cells (Figure 1C). The IC_50_ at which BFA induces cell death of K562 cells was 5.51 μM. The EC_50_ values by which BPF enhances the phosphorylation of MEK, ERK1, and ERK2 in K562 cells were 3.35 uM, 4.56 uM, and 4.58 uM, respectively.

**Figure 2:**
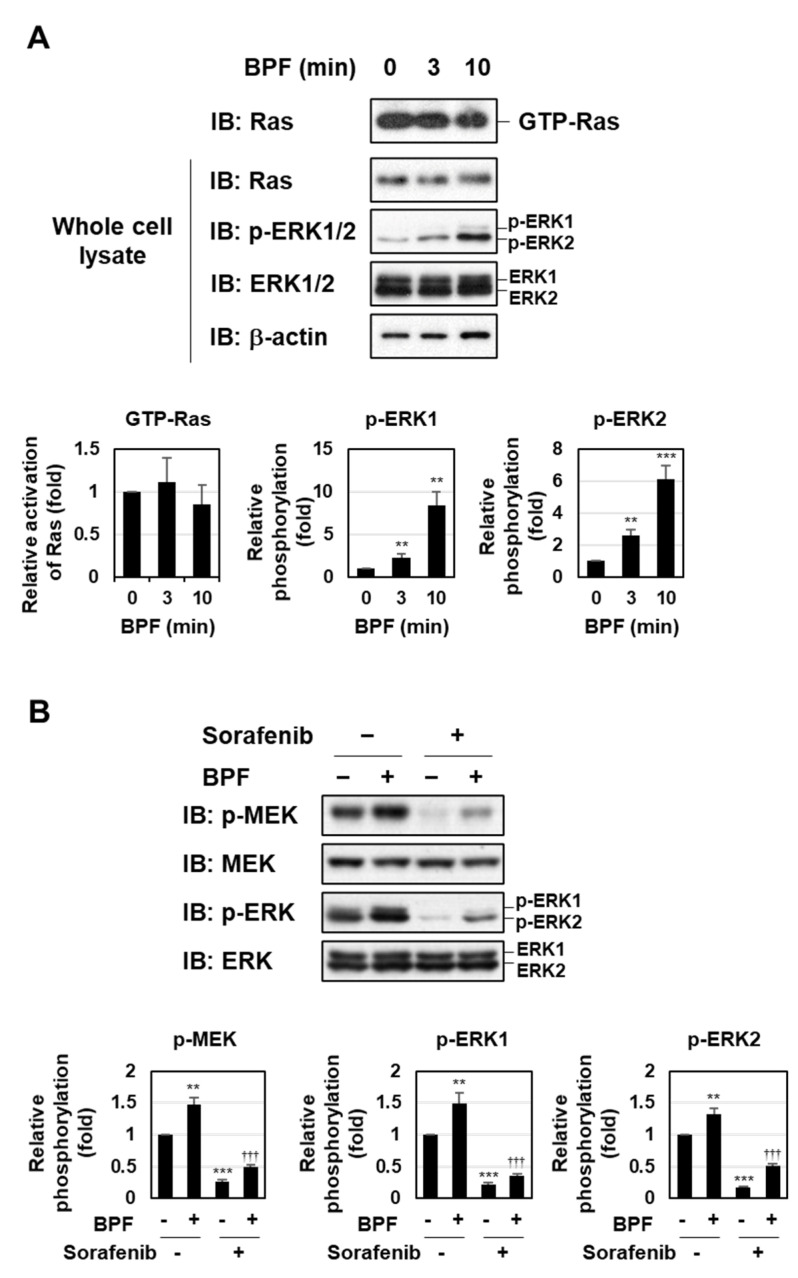
A bis-pyridinium fullerene derivative enhanced the activation of the MEK-ERK pathway through Raf, but not Ras, in K562 cells. (**A**) K562 cells were treated with BPF (10 μM) for the indicated periods, and cell lysates were prepared. The amount of activated Ras was measured by the affinity precipitation of Ras-GTP using GST-RBD, and immunoblotting was performed using an anti-Ras antibody. The expression of Ras and phosphorylation of ERK were assessed by immunoblotting. The relative activation of Ras and relative phosphorylation of ERK1 and ERK2 are shown in the graphs. (**B**) K562 cells were pre-treated with sorafenib (10 μM) for 1 h and then treated with BPF (10 μM) for 15 min. The phosphorylation and/or expression of MEK and ERK were assessed by immunoblotting. The relative phosphorylation levels of MEK, ERK1, and ERK2 are shown in the graphs. (**A**,**B**) Graphs show the mean ± SD. ** *p* < 0.01 and *** *p* < 0.001 indicate a significant difference from control cells. ^†††^ *p* < 0.001 indicates a significant difference from K562 cells treated with BPF alone.

To identify the intracellular signaling systems by which BPF activates the MEK-ERK pathway, we initially investigated whether BPF induced the activation of Ras, an upstream signaling molecule of the MEK-ERK pathway [7,8,9]. However, the activity of Ras in K562 cells was not affected by the treatment with BPF (Figure 2A). We then examined the involvement of Raf in the BPF-induced activation of the MEK-ERK pathway using the pan-Raf inhibitor sorafenib [21]. The treatment with sorafenib significantly inhibited the BPF-induced phosphorylation of MEK and ERK, suggesting that BPF enhanced the activation of the MEK-ERK pathway via Raf (Figure 2B).

### 2.2. BPF Induced Apoptosis by Enhancing the Activation of the MEK-ERK Pathway in K562 Cells

**Figure 3:**
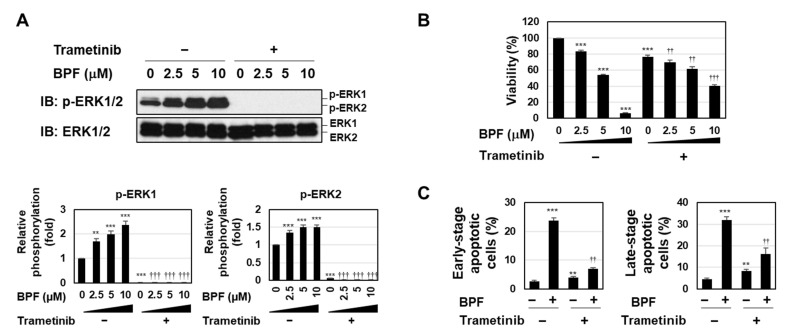
A bis-pyridinium fullerene derivative induced apoptosis through the enhanced activation of the MEK-ERK pathway. (**A**) K562 cells were pre-treated with trametinib (5 μM) for 1 h and then treated with BPF (2.5, 5, and 10 μM) for 6 h. The phosphorylation of ERK was assessed by immunoblotting. The relative phosphorylation of ERK1 and ERK2 is shown in the graphs. (**B**) K562 cells were pre-treated with trametinib (5 μM) for 1 h and then treated with BPF (2.5, 5, and 10 μM) for 24 h. Cell viability was measured by the WST assay (*n* = 4). (**C**) K562 cells were pre-treated with trametinib (5 μM) for 24 h and then treated with BPF (2.5, 5, and 10 μM) for 4 h. An annexin V/PI analysis was performed using flow cytometry (*n* = 3). (**A**–**C**) Graphs show the mean ± SD. ** *p* < 0.01 and *** *p* < 0.001 indicate a significant difference from control cells. ^††^ *p* < 0.01 and ^†††^ *p* < 0.001 indicate a significant difference from K562 cells treated with BPF alone.

Trametinib is a highly specific and potent MEK inhibitor [22]. To clarify the functional involvement of the MEK-ERK pathway in BPF-induced apoptosis in K562 cells, we analyzed the effects of trametinib on BPF-induced apoptosis. The treatment with trametinib inhibited the phosphorylation of ERK in untreated K562 cells and K562 cells treated with BPF (Figure 3A). The viability of trametinib-treated K562 cells was slightly reduced; however, the pretreatment with trametinib attenuated BPF-induced cytotoxicity, suggesting that the MEK-ERK pathway possesses bidirectional functions for cell survival (Figure 3B). Since we previously confirmed that BPF triggered apoptotic cell death [20], we performed an annexin-V/PI analysis in the present study. Although the treatment with trametinib slightly increased the population of early and late apoptotic cells in K562 cells, BPF-induced increases in the populations of early and late apoptotic cells were significantly reduced by the treatment with trametinib (Figure 3C). These results suggest that the BPF-induced activation of the MEK-ERK pathway was involved in the induction of apoptosis in K562 cells (Figure 3C).

### 2.3. BPF Induced the Activation of the MEK-ERK Pathway Independently of ROS Generation in K562 Cells

**Figure 4:**
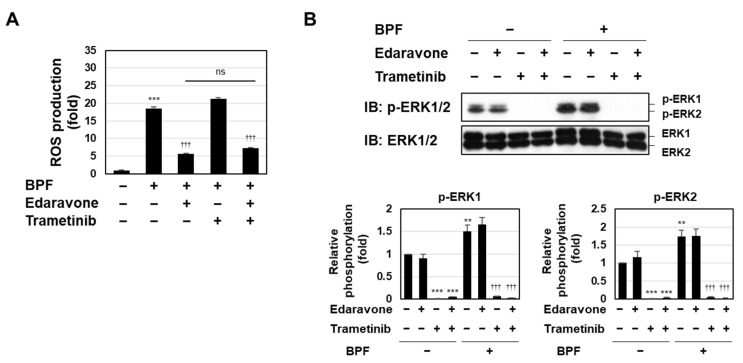
A bis-pyridinium fullerene derivative induced the activation of the MEK-ERK pathway in a ROS-independent manner in K562 cells. (**A**) K562 cells were pre-treated with edaravone (100 μM) and/or trametinib (5 μM) for 1 h and then treated with BPF (10 μM) for 30 min followed by a treatment with DCFH-DA (10 μM) for 30 min. Fluorescence in cells was measured (*n* = 4). (**B**) K562 cells were pre-treated with edaravone (100 μM) and/or trametinib (5 μM) for 1 h and then treated with BPF (10 μM) for 12 h. The expression and/or phosphorylation of ERK was detected by immunoblotting. The relative phosphorylation levels of ERK1 and ERK2 are shown in the graphs. (**A**,**B**) Graphs depict the mean ± SD. ** *p* < 0.01 and *** *p* < 0.001 indicate a significant difference from control cells. ^†††^ *p* < 0.001 indicates a significant difference from K562 cells treated with BPF alone. ns: not significant.

We subsequently investigated the relationship between ROS generation and the activation of the MEK-ERK pathway in BPF-induced K562 cell death using the potent free radical scavenger edaravone [23] and trametinib. Measurements of intracellular ROS levels using a DCFH-DA probe showed that they were strongly induced by BPF in K562 cells. Edaravone effectively suppressed BPF-induced ROS generation, whereas trametinib had no effects. In addition, the inhibition of ROS generation by edaravone was not affected by the co-treatment with trametinib (Figure 4A). The enhancement of MEK-ERK activation by BPF was prevented by the treatment with trametinib, but not edaravone (Figure 4B). These results suggest that BPF induced ROS generation and activated the MEK-ERK pathway in an independent manner in K562 cells.

### 2.4. BPF Induced Cell Death through Two Pathways, ROS Generation and the Activation of the MEK-ERK Pathway, in K562 Cells

**Figure 5:**
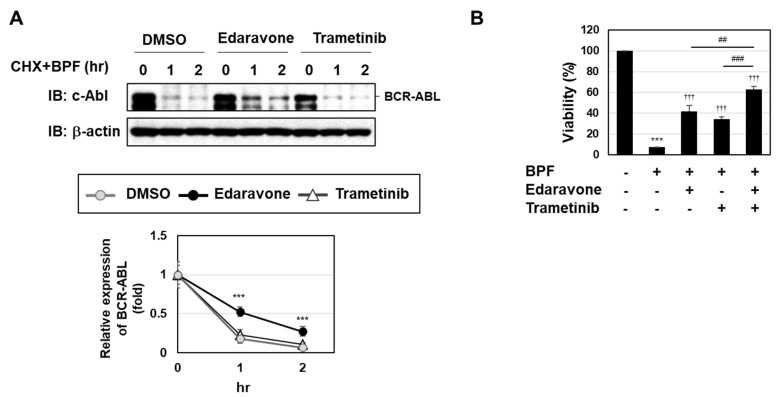
A bis-pyridinium fullerene derivative induced cell death through the generation of ROS and activation of the MEK-ERK pathway in K562 cells. (**A**) K562 cells were pre-treated with DMSO (0.1%), edaravone (100 μM), or trametinib (5 μM) for 1 h and then treated with BPF (10 μM) in combination with CHX (100 μg/mL) for the indicated periods. The expression of BCR-ABL and β-actin was detected by immunoblotting. The relative expression of BCR-ABL was shown in the graphs. Graphs show the mean ± SD. *** *p* < 0.001 indicates a significant difference from control cells. ^†††^ *p* < 0.001 indicates a significant difference from K562 cells treated with BPF alone. (**B**) K562 cells were pre-treated with edaravone (100 μM) and/or trametinib (5 μM) for 1 h and then treated with BPF (10 μM) for 24 h. Cell viability was measured using the WST assay (*n* = 4). *** *p* < 0.001 indicates a significant difference from control cells. ^†††^ *p* < 0.001 indicates a significant difference from K562 cells treated with BPF alone. ^##^ and ^###^ indicate *p* < 0.01 and *p* < 0.001, respectively.

We previously reported that BPF induced the degradation of BCR-ABL partially through ROS generation [20]. We herein investigated whether the enhanced activation of the MEK-ERK pathway was involved in the BPF-induced degradation of BCR-ABL. In the presence of the protein synthesis inhibitor cycloheximide (CHX), BPF markedly induced the degradation of BCR-ABL. This degradation of BCR-ABL was significantly prevented by the treatment with edaravone, but not trametinib (Figure 5A). These results suggest that the BPF-induced activation of the MEK-ERK pathway was not involved in the degradation of BCR-ABL. We also examined the effects of a co-treatment with edaravone and trametinib on BPF-induced K562 cell death. As shown in Figure 5B, the co-treatment with edaravone and trametinib attenuated BPF-induced K562 cell death significantly more than the treatment with edaravone or trametinib alone. Therefore, BPF appeared to induced cell death via two pathways, ROS generation and the activation of the MEK-ERK pathway. 

### 2.5. Enforced Expression of the BRAF^V600E^ Mutant Enhanced the Sensitivity of K562 Cells to BPF

**Figure 6:**
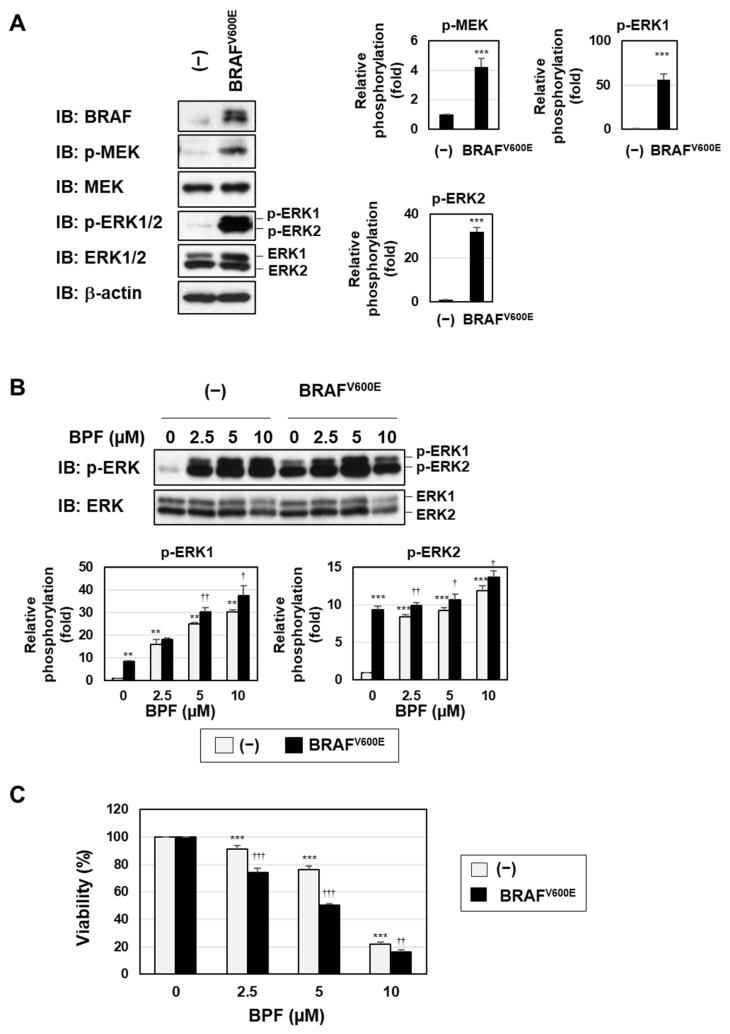
A bis-pyridinium fullerene derivative more potently induced cell death in K562 cells expressing BRAF^V600E^. (**A**–**C**) K562 cells were infected with an empty virus (-) or a virus expressing BRAF^V600E^. (**A**) Transduced cells were incubated with RPMI medium containing 1% FBS for 72 h. The expression and/or phosphorylation of BRAF, MEK, ERK, and β-actin were detected by immunoblotting. The relative phosphorylation levels of MEK, ERK1, and ERK2 are shown in the graphs. (**B**) Transduced cells were incubated with RPMI medium containing 1% FBS for 72 h and were then treated with BPF (2.5, 5, and 10 μM) for 6 h. The phosphorylation of ERK was detected by immunoblotting. The relative phosphorylation levels of ERK1 and ERK2 are shown in the graphs. (**C**) Transduced cells were incubated with RPMI medium containing 1% FBS for 72 h and then treated with BPF (2.5, 5, and 10 μM) for 24 h. Cell viability was measured using the WST assay (*n* = 4). (**A**–**C**) Graphs show the mean ± SD. ** *p* < 0.01 and *** *p* < 0.001 indicate a significant difference from control cells. ^†^ *p* < 0.05, ^††^*p* < 0.01, and ^†††^ *p* < 0.001 indicate a significant difference from control K562 cells treated with BPF alone.

To further investigate the role of the MEK-ERK pathway in BPF-induced cell death, we established K562 cells stably expressing a constitutively active mutant of BRAF (BRAF^V600E^), which was previously shown to be expressed in melanomas and induced the constitutive activation of its downstream MEK-ERK pathway [24]. The expression of BRAF^V600E^ induced the phosphorylation of MEK and ERK in K562 cells under serum-starved conditions (Figure 6A). The treatment with BPF increased the phosphorylation level of ERK in both control K562 cells and K562 cells expressing BRAF^V600E^ (Figure 6B). Although the viability of K562 cells was not affected by the expression of BRAF^V600E^ under serum-starved conditions, its expression significantly increased the sensitivity of cells to BPF (Figure 6C).

### 2.6. BPF Overcame Gatekeeper Mutation-Induced Resistance to BCR-ABL Inhibitors

**Figure 7:**
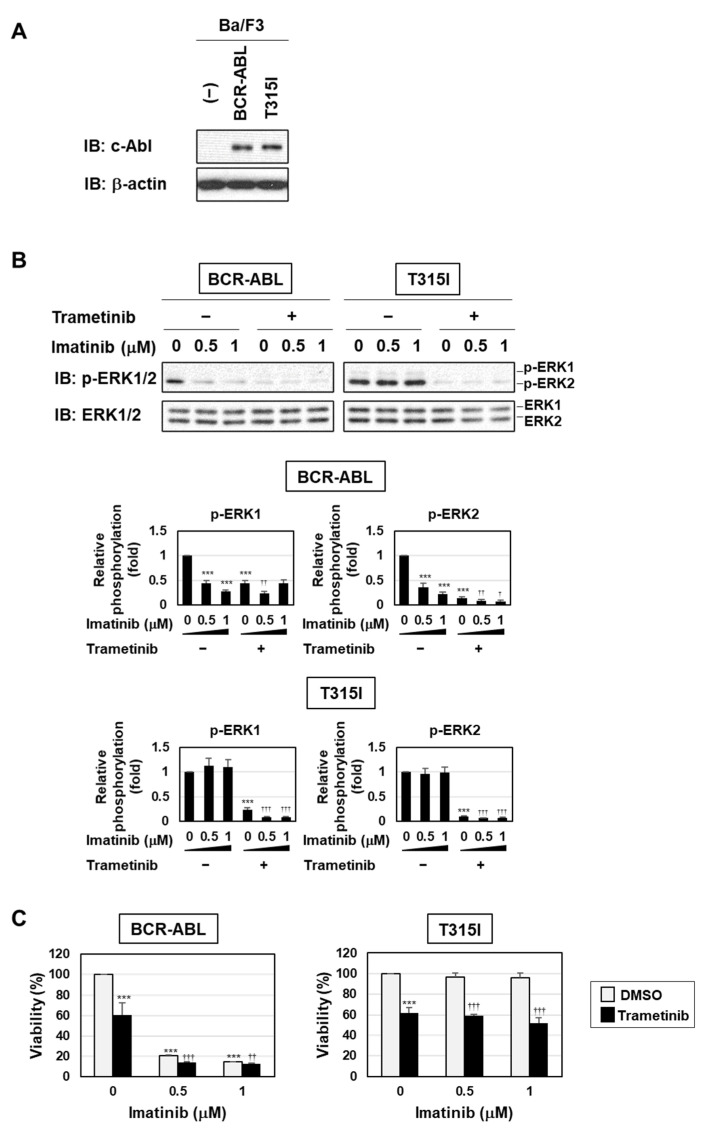
Imatinib inhibited the phosphorylation of ERK and induced apoptosis in Ba/F3 cells expressing BCR-ABL, but not Ba/F3 cells expressing the T315I mutant. (**A**–**C**) Ba/F3 cells were infected with an empty virus (-) or a virus expressing p190^BCR-ABL^ (BCR-ABL) and its point mutant (T315I). (**A**) The expression of BCR-ABL, the T315I mutant, and β-actin was detected by immunoblotting. (**B**) Ba/F3 cells expressing BCR-ABL or the T315I mutant were pre-treated with trametinib (5 μM) for 1 h and then treated with imatinib (0.5 or 1 μM) for 12 h. The phosphorylation of ERK was detected by immunoblotting. The relative phosphorylation of ERK1 and ERK2 is shown in the graphs. (**C**) Transduced Ba/F3 cells expressing BCR-ABL or the T315I mutant were pre-treated with trametinib (5 μM) for 1 h and then treated with imatinib (0.5 or 1 μM) for 24 h. Cell viability was measured by the WST assay (*n* = 4). (**B**,**C**) Graphs show the mean ± SD. *** *p* < 0.001 indicates a significant difference from control cells. ^†^ *p* < 0.05, ^††^ *p* < 0.01, and ^†††^ *p* < 0.001 indicate a significant difference from K562 cells treated with imatinib alone.

BPF has been shown to inhibit cell transformation by BCR-ABL with or without the gatekeeper mutation (T315I), partially through ROS generation [20]. Therefore, we herein examined whether BPF induced apoptosis in cells resistant to BCR-ABL inhibitors through the activation of the MEK-ERK pathway using Ba/F3 cells expressing BCR-ABL and the T315I mutant. The treatment with imatinib inhibited the phosphorylation of ERK in Ba/F3 cells expressing BCR-ABL, but not in Ba/F3 cells expressing BCR-ABL and the T315I mutant. On the other hand, the treatment with trametinib markedly inhibited the phosphorylation of ERK in both cells (Figure 7A,B). Imatinib induced apoptosis in Ba/F3 cells expressing BCR-ABL, but not in those expressing the T315I mutant, while trametinib reduced the viability of Ba/F3 cells expressing BCR-ABL and those expressing the T315I mutant. Furthermore, the co-treatment with trametinib enhanced imatinib-induced cell death in Ba/F3 cells expressing BCR-ABL, but not in those expressing the T315I mutant (Figure 7C).

**Figure 8:**
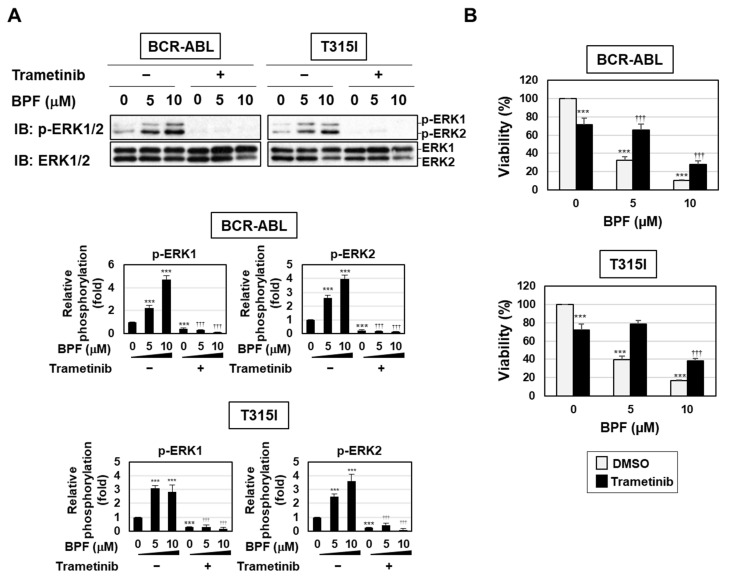
A bis-pyridinium fullerene derivative induced cell death through the activation of the MEK-ERK pathway in Ba/F3 cells expressing BCR-ABL or the T315I mutant. (**A**) Transduced Ba/F3 cells expressing BCR-ABL or the T315I mutant were pre-treated with trametinib (5 μM) for 1 h and then treated with BPF (5 or 10 μM) for 6 h. The phosphorylation of ERK was detected by immunoblotting. The relative phosphorylation of ERK1 and ERK2 is shown in the graphs. (**B**) Transduced Ba/F3 cells expressing BCR-ABL or the T315I mutant were pre-treated with trametinib (5 μM) for 1 h and then treated with BPF (5 or 10 μM) for 12 h. Cell viability was measured using the WST assay (*n* = 4). (**A**,**B**) Graphs show the mean ± SD. *** *p* < 0.001 indicate a significant difference from control cells. ^†††^ *p* < 0.001 indicates a significant difference from K562 cells treated with BPF alone.

On the other hand, BPF enhanced the phosphorylation of MEK and ERK in Ba/F3 cells expressing BCR-ABL and those expressing the T315I mutant, and this was inhibited by the treatment with trametinib (Figure 8A). BPF effectively induced cell death to a similar extent in Ba/F3 cells expressing BCR-ABL and those expressing the T315I mutant. In addition, BPF-induced cell death in both Ba/F3 cell types was significantly attenuated by the treatment with trametinib (Figure 8B). These results suggest that BPF induced cell death, even in imatinib-resistant cells, through the enhanced activation of the MEK-ERK pathway.

## 3. Discussion

**Figure 9:**
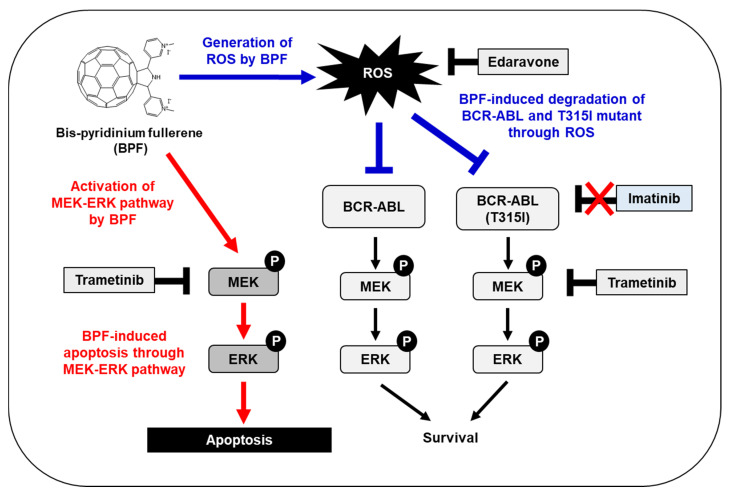
A bis-pyridinium fullerene derivative induces apoptosis through two individual pathways, ROS generation and the activation of the MEK-ERK pathway, in BCR-ABL-positive CML cells. BCR-ABL induces the activation of the MEK-ERK pathway, which is involved in cell survival. BCR-ABL 315I mutant exhibits resistance to imatinib. BPF induces the degradation of BCR-ABL and the T315I mutant through ROS generation. BPF also induces the activation of the MEK-ERK pathway, which induces apoptosis. Trametinib is a MEK inhibitor and edaravone is a scavenger of ROS.

We previously reported that BPF potently induced apoptosis in various cancer cells partially through ROS generation [19,20]. In the present study, we revealed the presence of a novel apoptotic pathway induced by BPF that was mediated by the MEK-ERK pathway in addition to ROS production (Figure 9). We also found that BPF targeted not only leukemia cells caused by BCR-ABL, but also leukemia cells expressing gatekeeper mutation-harbored BCR-ABL through these pathways (Figure 8B). Therefore, the mechanism underlying the anti-tumor activity of BPF was elucidated, and the results obtained suggest that BPF is an effective therapeutic agent for the treatment of CML.

Several studies have reported that fullerene derivatives accumulate to mitochondria and induce ROS production [25,26,27,28]. Combining our current observations [20] with these previous reports, we found that BPF causes apoptosis mediated by two individual pathways, ROS-mediated degradation of BCR-ABL and ROS-independent activation of MEK-ERK pathway. Therefore, ROS-mediated degradation of BCR-ABL seems to be due to the inhibitory effect on the mitochondrial transmembrane potential by BPF. On the other hand, we were not convinced that BPF-induced ERK activation is caused and mediated by mitochondrial functional alteration by BPF. This will be clarified in a future project.

We found that the MEK-ERK pathway activated by BPF was partially involved in BPF-induced apoptosis; however, the mechanisms by which BPF activates the MEK-ERK pathway currently remain unclear. Previous studies reported that a nanocrystalline fullerene (nano-C_60_) positively regulated the phosphorylation of ERK in glioma and hippocampal neural cell lines [29,30]. Harhaji et al. showed that the antioxidant *N*-acetylcysteine suppressed nano-C_60_-induced ERK activation as effectively as the selective MEK inhibitor PD90059, indicating that ROS were responsible for the nano-C_60_-induced activation of ERK [29]. However, since BPF reinforced the activation of the MEK-ERK pathway in a ROS-independent manner in K562 cells (Figure 4B), the mechanisms by which BPF activates the MEK-ERK pathway may differ from those by nano-C_60_. Although BPF had no effect on the activity of Ras, the BPF-induced phosphorylation of MEK and ERK was prevented by sorafenib, suggesting that BPF enhanced the activation of the MEK-ERK pathway by activating Raf, but not Ras, in K562 cells (Figure 2). Protein kinase C (PKC) has been shown to activate the Raf-MEK-ERK signaling pathway [31,32]. Although phorbol 12-myristate 13-acetate (PMA) and BPF both enhanced the phosphorylation of ERK in K562 cells, PMA, but not BPF, increased the activity of PKC (Figure S1). These results support the idea of BPF-induced activation of the MEK-ERK pathway not being mediated by PKC; however, the mechanism by which BPF activates the MEK-ERK pathway remains unclear. There is another possibility that BPF could activate Raf-MEK-ERK pathway through inhibiting its specific protein phosphatases. Until now, only the DUSP family is well known as a specific protein phosphatase for MAP kinases; however, other types of protein phosphatases for Raf and MEK kinases have not been identified yet [33]. We would like to clarify the detailed mechanism for BPF-caused ERK activation in future project.

The MEK-ERK pathway is generally considered to be involved in the proliferation and survival of numerous cells [34,35]. On the other hand, the role of the MEK-ERK pathway as a double-edged sword is also well documented and prolonged ERK signaling has been shown to initiate apoptosis [36]. As shown in Figure 3B and Figure 7C, the treatment with trametinib reduced the viability of K562 cells and Ba/F3 cells expressing BCR-ABL, suggesting that the MEK-ERK pathway activated downstream of BCR-ABL is essential for cell survival. Furthermore, the BPF-induced activation of the MEK-ERK pathway was sustained for at least 12 h (Figure 1C). The present results showed that BPF-induced cell death was prevented by the treatment with trametinib in both K562 cells and Ba/F3 cells expressing BCR-ABL (Figure 3B and Figure 7C). These results strongly suggest that the MEK-ERK pathway activated downstream of BCR-ABL and the MEK-ERK pathway activated by BPF have opposite functions. However, the mechanisms contributing to differences in the functions of the MEK-ERK pathway in the same cells have not yet been elucidated. Further studies to identify differences between the substrates of ERK in K562 cells and those of ERK in cells activated by BPF are needed to clarify the mechanisms responsible for cell survival and death via the MEK-ERK pathway.

At this stage, we have been not able to distinguish between the function of endogenous MEK-ERK activation in K562 cells and the function of BPF-induced activation of the MEK-ERK pathway. However, we need to consider about the effect on cell proliferation and survival of normal cells through ERK inhibition. As shown in Figure 1B, BPF induces the activation of both ERK1 and ERK2. It was reported that ERK2 is indispensable for mesoderm and placental development [37,38], suggesting that BPF could not be utilized for drug therapeutic treatment of pregnant patients. In addition, the several studies revealed that ERK2 contributes to the long-term memory, learning, and social behavior [39,40]. When using BPF, we need to care about the possibility that BPF could cause the side effects on the abilities of memory, learning and social behavior.

In summary, we herein demonstrated that BPF induced apoptosis in CML cells through two pathways: ROS generation and the enhanced activation of the MEK-ERK pathway (Figure 9). The BPF-induced generation of ROS contributed to the downregulation of BCR-ABL, whereas the BPF-induced activation of the MEK-ERK pathway did not affect the stability of BCR-ABL (Figure 5A). Therefore, BPF is expected to exhibit potent anti-tumor activity against various types of cancer cells other than CML through the activation of the MEK-ERK pathway.

## 4. Materials and Methods

### 4.1. Cell Culture and Retrovirus Infection

The CML-derived cell line K562 and murine pro-B cell line Ba/F3 were purchased from the Riken Cell Bank (Ibaraki, Japan). Ba/F3 cells expressing p190^BCR-ABL^ and its point mutant (T315I) were established as previously described [41]. K562 cells and transduced Ba/F3 cells were cultured in RPMI 1640 (Nacalai Tesque, Kyoto, Japan) supplemented with 10% fetal bovine serum (Biowest, Nuaillé, France), 100 units/mL penicillin (Nacalai Tesque), and 100 μg/mL streptomycin (Nacalai Tesque). K562 cells were infected with an empty virus and a retrovirus to express BRAF (V600E) using RetroNectin (Takara Bio Inc., Shiga, Japan), and infected K562 cells were selected using culture media supplemented with 2 μg/mL puromycin (InvivoGen, San Diego, CA, USA).

### 4.2. Measurement of Cell Viability

K562 cells, transduced K562 cells, and transduced Ba/F3 cells (5 × 10^4^ cells/100 μL) were seeded on 96-well plates using culture media. These cells were pre-treated with edaravone (100 μM) or trametinib (5 μM) for 1 h and then treated with the fullerene derivative BPF for 12 or 24 h. A water-soluble tetrazolium (WST) assay was performed using WST reagent (Nacalai Tesque, Kyoto, Japan), and cell viability was assessed as previously described [41].

### 4.3. Annexin V/PI Assay

K562 cells and Ba/F3 cells (5 × 10^5^ cells/2 mL) were seeded on 6-well plates. Cells were pre-treated with edaravone (100 μM) or trametinib (5 μM) for 1 h and then treated with BPF for 6 h. The induction of early and late-stage apoptosis was investigated using the annexin-V/PI assay (Nacalai Tesque) as previously reported [42].

### 4.4. Measurement of Intracellular ROS

After the treatment with BPF, cells were sedimented at 800× *g*, resuspended in phosphate-buffered saline (PBS) containing 10 μM DCFH-DA, incubated at 37 °C for 30 min in the dark, washed with PBS, and then seeded on 96-well black plates (Thermo Fisher Scientific, Waltham, MA, USA). The intensity of fluorescence was read at 480/530 nm (excitation/emission) using a microplate reader (Tecan Group Ltd., Männedorf, Switzerland).

### 4.5. Immunoblotting

K562 cells and Ba/F3 cells were pretreated with edaravone, trametinib, Gouml 6983, or sorafenib for 1 h prior to the treatment with BPF. Cell lysates were prepared, and immunoblotting was performed as previously described [41]. Band intensity was quantified using ImageJ software (National Institutes of Health, Bethesda, MD, USA).

### 4.6. Measurement of Ras Activity

Ras activity was measured using the Ras Activity Assay Kit (Cytoskeleton Inc., Denver, CO), following the manufacturer’s instructions. In brief, cell lysates were incubated with the Ras-binding domain of Raf-1 (Raf-RBD) coupled to agarose beads at 4 °C on a rotator for 1 h, and agarose beads were then collected by centrifugation and washed twice in wash buffer (25 mM Tris (pH 7.5), 30 mM MgCl_2_, 40 mM NaCl). GTP-bound Ras, which interacts with Raf-RBD, was detected by immunoblotting using the anti-Ras antibody.

### 4.7. Statistical Analysis

Data are shown as the mean ± SD. All experiments were repeated at least three times. A one- or two-way analysis of variance (ANOVA) followed by Tukey’s test was used to evaluate differences between more than three groups. A *p*-value < 0.05 was considered to be significant.

## 5. Conclusions

Our study demonstrated that BPF induces apoptosis in human CML-derived K562 cells via two independent pathways, ROS production and the enhancement of the activity of the MEK-ERK pathway. Through these two pathways, BPF was also shown to be effective against imatinib-resistant BCR-ABL mutant expressing cells. Therefore, BPF has potential as a therapeutic drug for CML as well as for various types of cancer cells mediated by aberrant activation of MEK-ERK pathway.

## Data Availability

Not applicable.

## Supplementary Materials

The following are available online at https://www.mdpi.com/article/10.3390/ijms23020749/s1.

## Abbreviations

Abl	Abelson
ALL	acute lymphocytic leukemia
Bcr	breakpoint cluster region
BPF	bis-pyridinium fullerene derivative
CHX	cycloheximide
CML	chronic myeloid leukemia
DCFH-DA	2′, 7′-dichlorodihydrofluorescein diacetate
ERK	extracellular signal-regulated kinase
FBS	fetal bovine serum
MEK	mitogen-activated protein kinase/extracellular signal-regulated kinase kinase
PBS	phosphate-buffered saline
Ph	Philadelphia chromosome
RBD	Ras-binding domain
ROS	reactive oxygen species
SOS	Son of Sevenless
STAT5	signal transducers and activator of transcription 5
WST	water-soluble tetrazolium
