# Supplementary material for: Novel Mechanism by a Bis-Pyridinium Fullerene Derivative to Induce Apoptosis by Enhancing the MEK-ERK Pathway in a Reactive Oxygen Species-Independent Manner in BCR-ABL-Positive Chronic Myeloid Leukemia-Derived K562 Cells
Source: Int J Mol Sci. 2022 Jan 11;23(2):749. doi: 10.3390/ijms23020749 (PMC8775703; doi:10.3390/ijms23020749)
Supplement: Supplementary file 1 [file ijms-23-00749-s001.zip › ijms-1536957-supplementary.pdf]

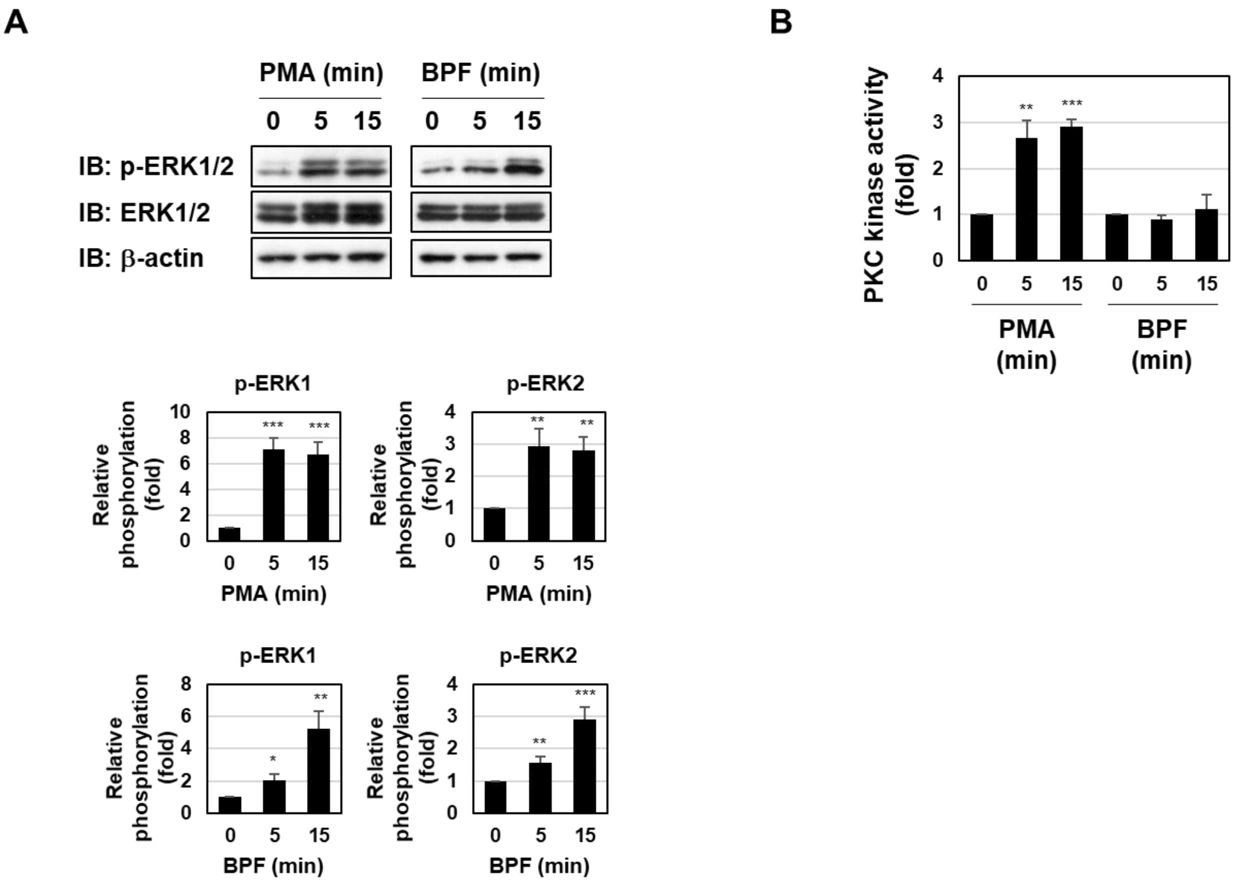

Supplemental Figure S1. BPF did not induce the activation of PKC in K562 cells. K562 cells were treated with PMA (100 nM)(Nacalai Tesque, Kyoto, Japan) or BPF (10 μM) for the indicated periods. (A) Cell lysates were immunoblotted with an anti-phospho-ERK antibody, anti-ERK antibody, or anti-β-actin antibody. The relative phosphorylation levels of ERK1 and ERK2 were shown in the graphs. Graphs depict the mean ± SD. $^{*}P<0.05$, $^{**}P<0.01$, and $^{***}P<0.001$ indicate a significant difference from control cells. (B) The activity of PKC was measured using a PKC assay kit (Abcam, Cambridge, UK) Graphs depict the mean ± SD. $^{**}P<0.01$ and $^{***}P<0.001$ indicate a significant difference from control cells.
